# Biomechanical and Morphological Analyses of Enamel White Spot Lesions Treated by Different Therapeutic Approaches (In Vitro Comparative Study)

**DOI:** 10.3390/dj13090408

**Published:** 2025-09-05

**Authors:** Lamis Abdul Hammed Al-Taee, Mohammad Talal Al-Hyazaie, Rabeia J. Khalil, Avijit Banerjee

**Affiliations:** 1 Department of Conservative and Aesthetic Dentistry, Baghdad College of Dentistry, University of Baghdad, Baghdad P.O. Box 0765, Iraq; mohammed.t@codental.uobaghdad.edu.iq; 2 Department of Restorative and Aesthetic Dentistry, College of Dentistry, University of Diyala, Baquba P.O. Box 78, Iraq; d.rabea779@uodiyala.edu.iq; 3 Centre of Oral Clinical Translational Sciences/Conservative & MI Dentistry, Faculty of Dentistry, Oral & Craniofacial Sciences, Kings’ College London, London WC2R 2LS, UK; avijit.banerjee@kcl.ac.uk

**Keywords:** bovine collagen, regenerate, Sylc air abrasion, CO_2_ laser, enamel white spot lesions, Raman spectroscopy, Knoop microhardness

## Abstract

**Background/Objectives**: Within the minimum intervention oral care (MIOC) delivery framework, the management and improvement in the esthetics of enamel white spot lesions (WSLs) are recommended. This study evaluated the chemomechanical and morphological characteristics of WSLs treated by four therapeutic approaches using Raman spectroscopy, Knoop microhardness (KH), and field-emission scanning electron microscopy (FESEM). **Methods**: Sixty human enamel slabs were divided into six groups: non-treated (baseline), WSLs (8% methylcellulose gel with 0.1 M lactic acid, pH 4.6 at 37 °C for 21 days), and four treated groups, namely bovine collagen supplement (Nutravita Ltd., Maidenhead, Berkshire, UK), Regenerate system (NR-5, Bordeaux, France), Sylc air abrasion (AquaCare, Denfotex Research Ltd., Edinburgh, UK), and CO_2_ laser (JHC1180, Jinan, China). Treatment lasted 28 days, followed by four weeks of storage in artificial saliva (pH = 7.0, 37 °C). Bovine collagen was analyzed using Fourier-Transform Infrared Spectroscopy (FTIR). The mineral content, including the phosphate peak intensities (PO_4_ ν_1_, ν_2_, and ν_4_) and carbonate (CO_3_), as well as tissue microhardness, were assessed at varying depths (50–200 µm), followed by morphological assessment. **Results**: The FTIR spectrum of bovine collagen powder confirms the presence of amide I, II, and III. It produced a statistically significant enhancement in the phosphate content and KHN compared to WSLs of up to 150 µm in depth (*p* < 0.001). Regenerate-treated surfaces recorded the highest phosphate content among groups at the superficial layer. All treatment interventions enhanced the morphology of lesions by covering the exposed prisms and inter-prismatic structure. **Conclusions**: Bovine collagen supplements can enhance the phosphate content and surface properties of enamel white spot lesions (WSLs) and could be considered a potential modality comparable to other micro-invasive approaches for addressing incipient enamel lesions. This could significantly impact dental care management.

## 1. Introduction

White spot lesions (WSLs) signify the initial phase of enamel demineralization following exposure to acids in the carious process; they slowly dissolve minerals below the relatively intact outer enamel surface, contributing to the development and progression of carious lesions [1]. This process is initiated by the breakdown of proteins followed by a depletion of inorganic ions within the inter-prismatic and intercrystalline areas [2]. Although saliva plays an essential role in maintaining the equilibrium between the demineralization and remineralization of enamel through its buffering and cleansing properties and ion saturation, its effectiveness is constrained by fluctuations in the pH of the oral environment [3]. Therefore, providing an external source of these ions can enhance the remineralization process and limit further structural damage in susceptible patients. This can be achieved by applying bioactive substances that supply phosphate, calcium, and fluoride ions, which form deposits that are potentially integrated into porous enamel surfaces. This encompasses the formation of new hydroxyapatite (HAp) crystals, the repair of existing crystals, and the deposition of external minerals [4].

Regenerate Enamel Science (NR-5, Bordeaux, France) is a dual gel phase system based on a combination of calcium silicate and sodium phosphate salts with fluoride. This system is proposed in order to augment the natural mineralization process of saliva by transforming the calcium silicate into Hap, which deposits on acid-depleted enamel surfaces [5]. This helps in repairing the demineralized enamel surfaces and protects them from acid damage. A previous study [6] reported higher phosphate levels and microhardness values for sound enamel surfaces treated with the Regenerate system, which enhanced their resistance to acid challenges. This treatment can induce the formation of a biomimetic firm layer that integrates into the surface, restoring the structure and morphology of the natural enamel’s hydroxyapatite [6,7].

Sylc (Denfotex Research Ltd., Inverkeithing, UK) offers a proactive and micro-invasive method for treating early carious lesions. It utilizes bioactive glass particles (45S5) that effectively penetrate and fill the microporosities in white spot lesions (WSLs). Bioactive glass (BAG) can form hydroxycarbonate apatite (HCA) when it comes into contact with an aqueous environment, along with the formation of a calcium and phosphate-dense layer that precipitates and chemically bonds to the demineralized surfaces, thus improving the characteristics of treated enamel surfaces [8,9].

Recently, as part of the minimum intervention oral care (MIOC) delivery framework, there has been a shift in managing WSLs, moving away from a fundamental invasive restorative strategy and towards micro-invasive enamel tissue regeneration to enhance the mechanical strength and tissue resilience [10]. This process aims to replicate the natural remineralization process of early enamel lesions instead of sealing them with substitute materials. This is achieved by using collagen-containing materials that are capable of forming a biomimetic scaffold aid in the deposition of minerals to support natural tissue remineralization by saliva [11,12]. Previous studies [12,13] have confirmed the ability of self-assembling peptides P11-4 (Curodont Repair, Vardis, Switzerland) to organize into complex structures that mimic the natural enamel matrix, aiding in tissue regeneration and remineralization. Bovine collagen supplements are derived from the collagen in the skin, bones, and cartilage of cows. They supply the essential peptides that support the regenerative process of bone formation and help in tissue repair [14]. This is due to the high collagen level, primarily type I collagen, which provides a framework for bone mineralization and enhances the mineral density, thus playing a critical role in bone remodeling. Although enamel lacks cellular regenerative capacity, our hypothesis is that collagen peptides may serve as a scaffold for mineral nucleation and enhance natural remineralization. This type of collagen has not yet been explored in the management of enamel caries; thus, it is proposed as a novel treatment approach for enamel repair.

A carbon dioxide (CO_2_) laser can be used for WSL management due to the photothermal effects that cause the melting and recrystallization of HAp crystals associated with a reduction in carbonate content. This reduces the solubility of enamel, making it more resistant to further acid dissolution [6,15]. The application of a CO_2_ laser at a 10.6 µm wavelength is recommended since it penetrates deeper into the enamel without harming the surface or raising the temperature of the dental pulp. Additionally, this wavelength is well-absorbed by the phosphate, carbonate, and hydroxyl groups in the hydroxyapatite [16]. Previous studies [17,18] have confirmed the ability of CO_2_ lasers to improve the hardness of softened enamel with a modest esthetic enhancement.

Raman spectroscopy is a non-invasive method that can quantitively measure the mineral distribution within dental hard tissues and detect the structural changes through their unique molecular vibrational energy signatures. This helps in evaluating the demineralization and remineralization of tooth enamel [6,19]. Additionally, surface microhardness is regarded as a direct indicator of the mineral content in dental tissues and can quantitively measure the amount of mineral loss or gain [6,20]. Accordingly, the present study evaluated and compared the chemomechanical and morphological characteristics of enamel WSLs treated by a bovine collagen supplement, the Regenerate system, Sylc- BAG air abrasion, and a carbon dioxide (CO_2_) laser. Initially, FTIR was conducted to examine the presence of amides that are needed to support the functionality of bovine proteins without any interference with other additives and ingredients. The assessments were carried out by using Raman spectroscopy, cross-sectional Knoop microhardness (KH) through the entire depth of treated WSLs (50–200 µm), and field-emission scanning electron microscopy (FESEM). The null hypothesis was that there are statistically no significant differences in Raman mineral peak intensities and KHN between non-treated enamel surfaces (baseline and WSLs) and treated surfaces at each layer and through the depth of the lesions.

## 2. Materials and Methods

### 2.1. Fourier Transform Infrared Spectroscopy (FT-IR) Analysis of Bovine Collagen

The ART/FTIR analysis was conducted with an IR Affinity-1S instrument (SHIMADZU, Kyoto, Japan), featuring a resolution of 1 cm^−1^, and data processing was accomplished using LabSolutions CS control software (version number A104123456). The infrared spectrum of the powder was recorded over the spectral range of 4000 to 400 cm^−1^ (*n* = 3).

### 2.2. Sample Size

The sample size was determined using G-Power 3.0.10 software, created by Franz Faul at the University of Kiel, Germany [21], with a research power set at 95%, a two-sided alpha level of 0.05 (two-tailed), and an effect size of F = 0.4 (considered a large effect); the required sample size for six groups and four measurements is approximately 60 samples, resulting in 10 samples per group, totaling 480 data points.

### 2.3. Specimen Preparation

Ten permanent sound human premolars were obtained from patients under 20 that had been extracted for orthodontic purposes in agreement with the ethical standards of the Research Ethics Committee with ethical approval number 617, 2 June 2023. These specimens were stored in deionized water in a refrigerated cabinet (+4 °C) for six weeks, which was refreshed weekly. The roots were cut and removed at the Cemento-Enamel Junction (CEJ) using an Isomet 1000 (Buehler, Lake Bluff, IL, USA). Each crown was split mesiodistally into two sections. The buccal halves were further sectioned into six slabs measuring 3.0 mm height × 3.0 mm width × 2.0 mm thickness, which were then included in epoxy resin molds. The slabs (*n* = 60) were assigned to six groups (*n* = 10 per group): baseline: sound enamel surface did not receive any treatment; WSLs left without treatment; collagen (WSLs immersed in bovine collagen supplement); Regenerate (WSLs treated with Regenerate toothpaste/serum); Sylc (WSLs subjected to Sylc air abrasion); and CO_2_ laser (WSLs subjected to CO_2_ Laser). Then all groups were stored in artificial saliva (1.5 mM CaCl_2_ × 2H_2_O, 0.9 mM KH_2_PO_4_, 130.0 mM KCl, 20.0 mM HEPES, 3.1 mM NaN_3_, adjusted to pH 7.0 with KOH) [22] for 28 days at 37 °C.

### 2.4. Artificially Induced WSLs

To remove the outer enamel surface, sequential polishing (Laryee Technology Co., Ltd., Beijing, China) was performed using a 500-grit silica carbide disk for five secs (Versocit, Struers A/S, Copenhagen, Denmark), 1200-grit for ten secs, 2500-grit for thirty secs, and, finally, 4000-grit for 4 min. This was followed by 10 min ultrasonication to gain flat and smooth surfaces. The lesion was created by using 8% methylcellulose gel (Sigma-Aldrich, Saint Louis, MO, USA) buffered with 0.1 M lactic acid (800 mL, AnalaR, Leicestershire, UK), and the pH was adjusted to 4.6 using 1 M of NaOH. Specimens were left in the demineralizing solution for 21 days at 37 °C, which was changed on weekly basis [23].

### 2.5. Bovine Collagen Supplement Application

According to the manufacturer’s guidelines, the maximum daily consumption of the bovine collagen supplement is 20 g (Pure Collagen Hydrolysate Power, 100% Pure Active Bovine Collagen Peptides, Nutravita Ltd., Maidenhead, UK). Two level scoops of the powder were mixed with 250 mL of water until homogenous. Then, samples were submerged in the solution for 2 min to facilitate the diffusion and self-assembly of the peptide on the WSLs. This process was repeated daily for four weeks, after which the specimens were stored in artificial saliva at 37 °C for four weeks. 

### 2.6. The Application of Regenerate Enamel Science System

The Regenerate system (Regenerate Enamel Science, NR-5 company, Bordeaux, France) consists of a serum, an activator, and toothpaste. The serum and toothpaste share a set of components, which include glycerin, calcium silicate, PEG-8, trisodium phosphate, sodium phosphate, water, sodium monofluorophosphate, hydrated silica, synthetic fluorphlogopite, sodium saccharin, and polyacrylic acid. Meanwhile, the activator gel is composed of glycerin, cellulose gum, sodium fluoride, benzyl alcohol, ethylhexylglycerin, phenoxyethanol, water, and sodium fluoride at a concentration of 1450 ppm. Following the manufacturer’s guidelines, the serum was applied to the surface for three minutes over three consecutive days. After that, a freshly prepared toothpaste slurry (4 mL per specimen) was blended with distilled water for 4 min until a uniform consistency was achieved at a 1:3 dilution ratio. The specimens were immersed in the mixture for 2 min every day for 28 days, after which the treated surfaces were preserved in artificial saliva at 37 °C for four weeks [6,24]. 

### 2.7. Sylc Air Abrasion

Sylc powder was propelled onto the demineralized surfaces using an air abrasion handpiece (AquaCare, VELOPEX International, Denfotex Research Ltd., London, UK). The Sylc powder (25–120 µm particle size) consists mainly of 45S5 bioactive glass, which is a calcium sodium phosphosilicate, combined with other ingredients (silicon 21%, calcium 18%, sodium 18%, phosphorus 3%, and oxygen 40%). This device operated at an air pressure of 40–46 psi, with a nozzle angle of 90°, and maintained a distance of 5 mm from the surface while performing circular movements, in accordance with the manufacturer’s guidelines. Each specimen received treatment for 10 s, after which the powder was retained for 2 min before being rinsed under running water [8,9], followed by four weeks of storage in artificial saliva at 37 °C, which was refreshed daily. 

### 2.8. Carbon Dioxide Laser

**Figure 1:**
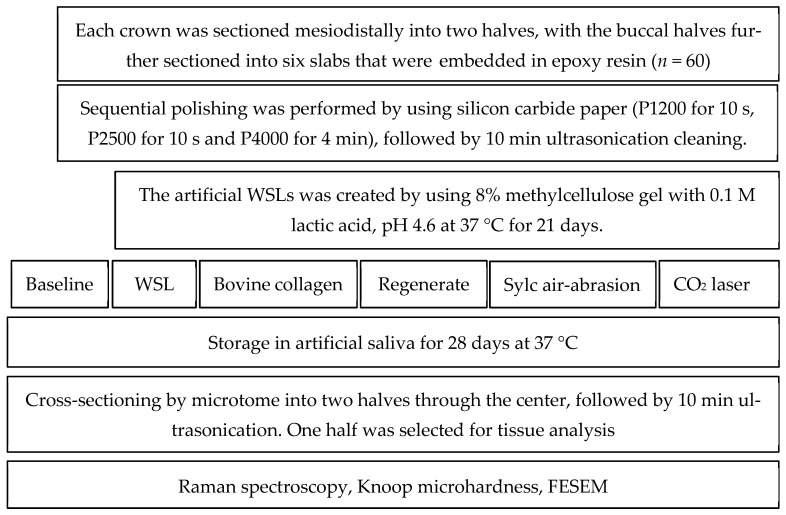
A flowchart summarizing the experimental workflow of this study.

A commercially available CO_2_ fractional laser (CO_2_ Fractional Laser, JHC1180, Jinan, China) was applied to the demineralized enamel surfaces with the following settings: a power output of 2 W, a pulse duration of 10 milliseconds, a pulse frequency of 50 Hz, and a 0.2 mm focal spot with an energy output of 11.5 J/cm^2^. The surface scanning operation lasted for 5 s and was conducted in a non-contact manner from an X–Y positioning platform, maintaining a distance of 10 mm between the handpiece tip and the enamel surface [6]. Water cooling was used during the procedure to simulate clinical conditions. After treatment and storage, each slab (*n* = 60) was hemi-sectioned into two halves through the center and ultrasonically cleaned for 10 min, with one half being selected for tissue analyses. The experimental procedures are summarized in Figure 1.

### 2.9. Chemical Characterization (Raman Spectroscopy)

**Figure 2:**
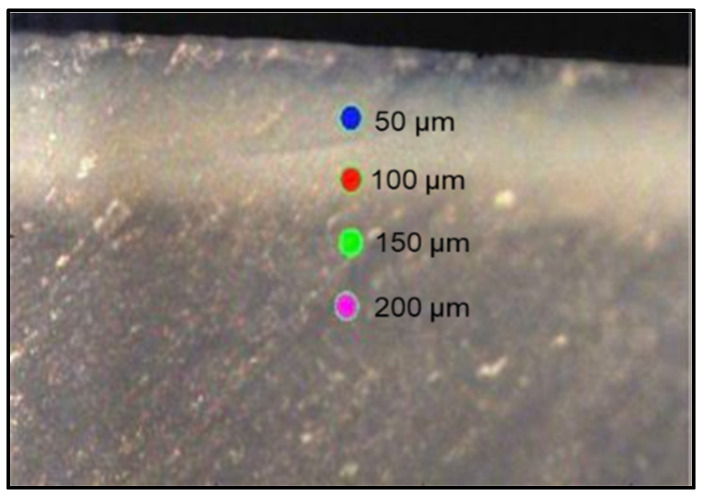
A representative enamel slab illustrates the designated points analyzed by a high-resolution confocal Raman spectroscope (785 nm near-infrared diode laser and a 400 line/mm diffraction grating); an Olympus 20×/0.40 NA objective lens (Olympus, Tokyo, Japan) was used to focus the laser on the sample surface with a spot size of about 5 μm.

A total of 240 points were scanned across the hemi-sected surfaces of 60 enamel slabs (four measurements per slab) using high-resolution confocal laser Raman spectroscopy (Senterra, Bruker Optics, Ettlingen, Germany). This system utilized a 780 nm near-infrared diode laser and a grating of 400 lines/mm. The spectra were recorded over a frequency range of 100–3800 cm^−1^, employing a laser power of 100 mW and an integration period of 30 s for each measurement point. The scan points were positioned 50 μm apart, as illustrated in Figure 2. Following the acquisition and processing of spectra (including baseline correction) with Raman software (OPUS, Bruker Optics, Germany), four distinct Raman spectroscopic peaks were observed. The intensities of the phosphate peaks ν_1_, ν_2_, and ν_4_ were recorded at 960, 433, and 579 cm^−1^, respectively, and the carbonate peak was noted at 1070 cm^−1^. The Raman spectroscope was operated in point-scanning mode, examining the sample from the surface down to a depth of 50–200 µm.

### 2.10. Cross-Sectional Microhardness

The hardness profile was assessed by a Knoop microhardness tester (HST-1000 microhardness tester; Jinan Hensgrand Instrument Co., Ltd., Jinan, China), utilizing a diamond-shaped rhombohedral indenter that applied a 25 gf load for 10 s [25]. A total of 240 indentations (*n* = 4 per sample) were performed at the same points previously examined with micro-Raman spectroscopy (Figure 2). The Knoop hardness number was automatically recorded through the software provided by the manufacturer. 

### 2.11. Morphological Assessment by FESEM

The morphology of the lesions and the treated surfaces was visualized using a digital microscope (Bysameyee, USB Digital Microscope, 40×–1000× Magnification Endoscope, 8 LED Mini Video Camera, Yerevan, Armenia). Two samples from each group were gold-coated and examined using a field-emission scanning electron microscope (Inspect F50, FEI Company, Eindhoven, The Netherlands). The analysis was conducted at an accelerating voltage of 30 kV, utilizing three magnification levels for surface imaging (2500, 5000, and 10,000×) and cross-sectional views (600, 1300, and 5000×).

### 2.12. Statistical Analyses

Statistical analyses were conducted using SPSS version 26.0 (SPSS, Inc., an IBM Company, Chicago, IL, USA). To assess the equality of variances and the distribution of data, the Shapiro–Wilk test was used. Since the data met the criteria for equal variances and normal distribution, a multivariate ANOVA followed by Tukey’s post hoc multiple comparisons was performed to evaluate the differences in the intensity of Raman mineral peaks (A.U.) and Knoop microhardness number (KHN) among groups at each layer and between layers (*p* < 0.05). 

## 3. Results

### 3.1. FTIR Spectral Analysis

**Figure 3:**
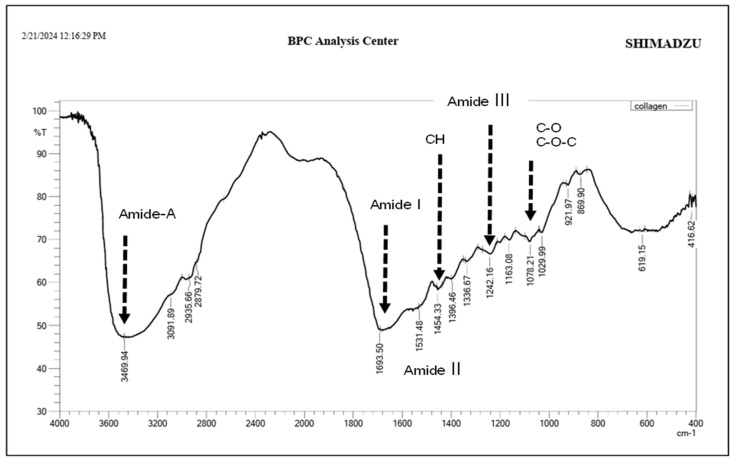
FTIR spectrum of bovine collagen powder.

FTIR spectra of bovine collagen powder confirm the presence of ν (C=O) absorption of amide I at 1693.50 cm^−1^ and amide II at 1531.48 cm^−1^ and ν (C-N) *δ*(CH_2_) and *δ*(CH_3_) absorptions in the range of 1350–1480 cm^−1^, as well as *δ*(N-H) absorptions of amide III at 1242.16 cm^−1^. Additionally, ν (C-O) and ν (C-O-C) absorptions of carbohydrate moieties at 1078.21 cm^−1^ are observed [26]. The amide-A band (NH stretching) was noted at 3469.94 cm^−1^, as shown in Figure 3.

### 3.2. Chemical Analysis (Raman Results)

The Raman results showed that the phosphate ν_1_ peak intensity increased in all surfaces from the superficial layer to the deeper layers; however, this increase was statistically significant only in the demineralized and collagen-treated groups (*p* < 0.001). The enamel-treated surfaces exhibited a statistically significant higher mean intensity of phosphate ν_1_ (*p* < 0.001) than the WSLs up to a depth of 150 µm. Beyond that depth, the mean intensity was comparable across all groups (*p* > 0.05). At the superficial layer (50 µm), the Regenerate-treated surfaces demonstrated the highest PO_4_ ν_1_ intensity among the groups, which was statistically significant compared to both the baseline and collagen-treated surfaces (*p* = 0.035 and 0.045, respectively). However, in the deeper layers (>100 µm), the differences between the treated and baseline surfaces were not statistically significant (*p* > 0.05), as shown in Table 1. Similarly, the mean intensity of the phosphate ν_2_ peak increased in the deeper layers across all groups; however, the difference was not statistically significant in the Sylc- and laser-treated groups (*p* = 0.051 and 0.490, respectively). The WSL group recorded the lowest phosphate ν_2_ peak intensity among groups throughout the entire depth of the lesion (50–200 µm). At the superficial layer (50 µm), the Regenerate-, Sylc-, and CO_2_ laser-treated surfaces exhibited the highest phosphate ν_2_ intensity compared to the collagen-treated group, which was comparable to the baseline (*p* = 1.000). The Regenerate-treated surfaces showed a statistically significant increase in mean intensity at both 100 and 150 µm depths; however, at the deeper layer (200 µm), the intensity was comparable to that of all groups (*p* > 0.05). There was a similar intensity among the collagen, Sylc, and laser-treated surfaces at depths ≥ 100 µm, (*p* > 0.05). The mean value of ν_4_ peak intensity also increased towards the deeper layers in all groups (*p* < 0.001). These values were statistically significantly higher in all treated surfaces than in the demineralized surfaces throughout the entire depth of the lesions (50–200 µm). The collagen-treated surfaces showed a comparable mean value to the baseline throughout the lesion depth, as well as to the laser and Sylc-treated surfaces in the deeper layers (100–200 µm). The Regenerate-treated surfaces recorded the highest ν_4_ peak values (*p* < 0.05) among all groups; however, the value was comparable to the baseline and all treated surfaces at 200 µm (*p* > 0.05).

The mean carbonate intensity was comparable between layers in all groups (*p* > 0.05), except for the laser- and Regenerate-treated surfaces (*p* = 0.005 and *p* < 0.001, respectively), which exhibited the highest carbonate content (*p* < 0.05) among the groups up to a depth of 150 µm. The laser treatment significantly reduced the mean carbonate intensity in the superficial layer (50 µm); however, it was comparable to the baseline and collagen-treated surfaces at a depth of 100 µm and all groups beyond 150 µm. Although the carbonate content in WSLs was higher than the baseline, collagen-, and Sylc-treated surfaces, this difference was not statistically significant (*p* > 0.05) throughout the lesion depth (50–200 µm) (Table 1).

### 3.3. Knoop Microhardness

**Figure 4:**
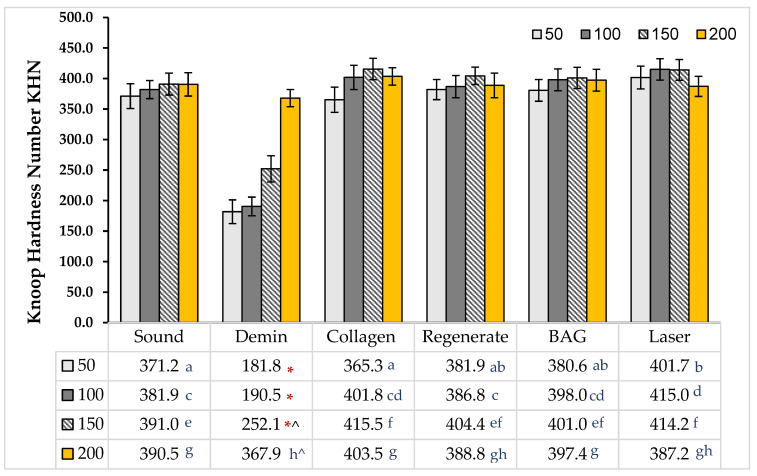
Mean KHN values of the baseline, WSL, collagen-, Regenerate-, Sylc-, and CO_2_-treated surfaces. Similar letters indicate that there were no statistically significant differences between treated surfaces at each depth. (*) The white spot lesions (WSLs) exhibited the lowest KHN among the groups (*p* < 0.05) at each layer up to a depth of 150 µm. (^) There were statistically significant differences in the values of each group between layers.

There was an increase in tissue hardness from the outermost layer towards the deeper layers in all groups, except for the demineralized surfaces (*p* < 0.05). At the superficial layer (50 µm), the laser-treated surfaces exhibited the highest KHN, which was not statistically significant different from that of the Regenerate- and Sylc-treated surfaces (*p* = 0.801 and 0.872, respectively). However, the mean values for both treatments were close to those of the baseline and collagen-treated surfaces (*p* > 0.05). The laser-treated surfaces maintained higher mean values in the deeper layers (100 and 150 µm), which were not statistically different from the KHN values of the collagen- or Sylc-treated groups (*p* > 0.05) but were significantly greater than those of the sound and demineralized groups (*p* < 0.05). At a depth of 200 µm, the collagen-treated surfaces recorded the highest KHN, which was comparable to the baseline and all treated surfaces but higher than that of the demineralized group (*p* < 0.001; Figure 4).

### 3.4. The Morphological Assessments

**Figure 5:**
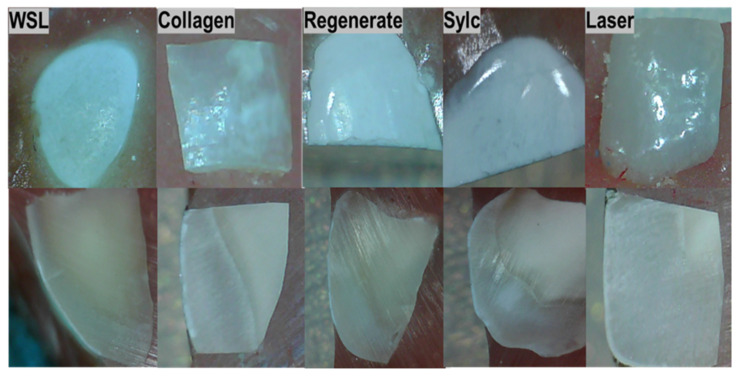
Photographs illustrating the morphological characteristics of WSLs (white spot lesions) at the surface and cross-section after treatment with four different interventions.

In the cross-sectional view, there is a continuous white line of demineralized area along the outer edge of the lesion. Treating the surface with bovine collagen enhances the appearance of the lesion, where areas of complete tissue repair interrupt the lesion in both surface and cross-section views. The mineralization of enamel is clearly observed in the Regenerate- and Sylc -treated surfaces, as there is a continuous layer of minerals covering the lesion, which reduces its opacity and restores a glossy appearance. There is a significant improvement in the appearance of WSLs when exposed to a CO_2_ laser, as the treated surface appears glossy, smooth, and more polished, with a recognizable reduction in the opacity of WSLs (Figure 5). 

**Figure 6:**
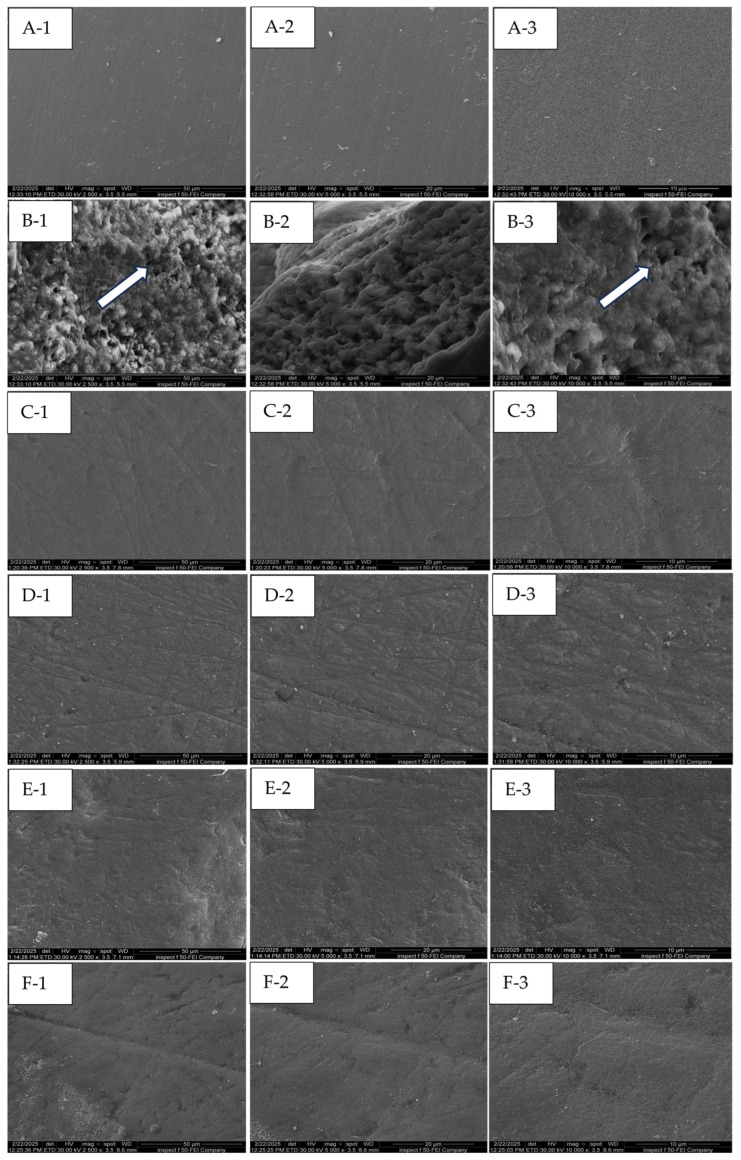
FE-SE micrographs show the surface morphology of the baseline, WSL, bovine collagen-, Regenerate-, Sylc-, and CO_2_ laser-treated surfaces (**A**, **B**, **C**, **D**, **E**, and **F**, respectively). The white arrow points to the honeycomb-like structure in the WSL, which is due to prism exposure and the distinct loss of inter-prismatic substances.

**Figure 7:**
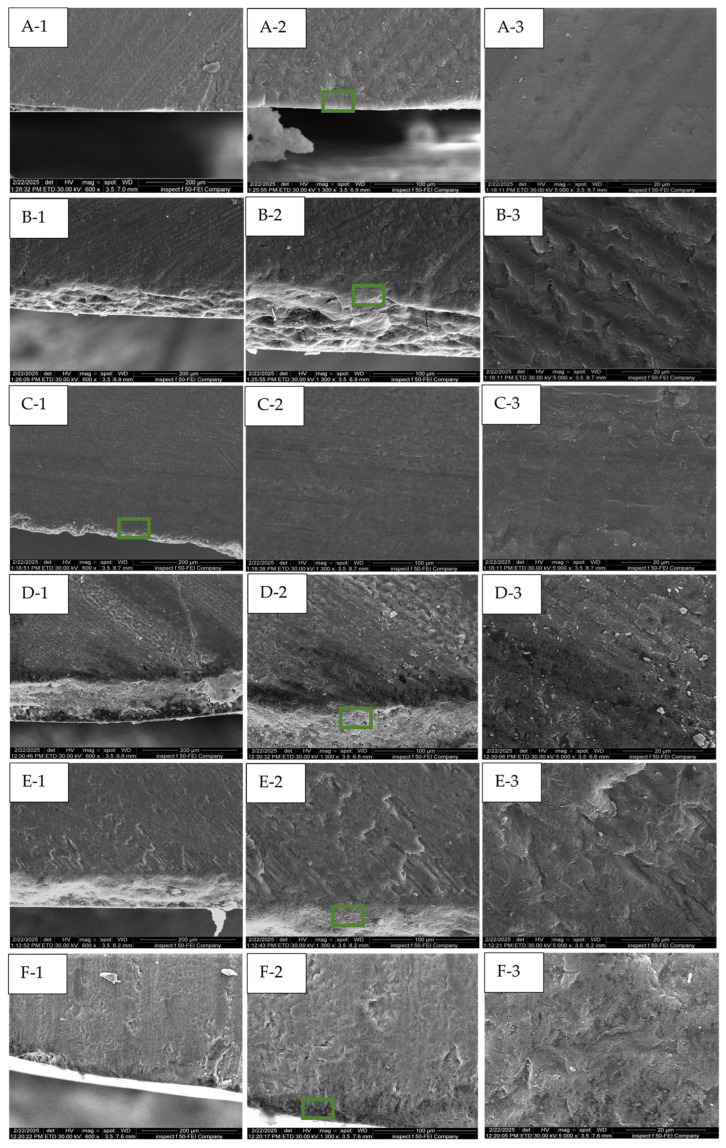
FE-SE micrographs illustrate the cross-sectional views of the baseline, WSL, bovine collagen-, Regenerate-, Sylc-, and CO_2_ laser-treated surfaces (**A**, **B**, **C**, **D**, **E**, and **F**, respectively). The green box highlights the area that is further magnified.

In the FESE micrographs, the baseline exhibits the characteristic features of an intact enamel surface, which appears smooth, polished, and free from surface defects at a magnification of 2500× to 10,000× (Figure 6(A-1–A-3)). In the cross-sectional view (Figure 7(A-1–A-3)), the enamel prisms reveal distinct bands with well-defined boundaries between the prisms and surrounding smooth areas. In WSLs (Figure 6B), a honeycomb-like structure is clearly observed (white arrow) due to prism exposure and increased surface porosity, accompanied by a distinct loss of the inter-prismatic substances. Similarly, in the cross-sectional view, there is a disorganized, rough surface associated with porosities, grooves, and scratches (Figure 7(B-3)). In the surface view, the lesions treated with bovine collagen, Regenerate, Sylc air abrasion (Figure 6: C, D, and E, respectively) were completely restored, with the prismatic structures and inter-prism spaces no longer distinct. In the Sylc group, the surface appears smoother than those treated with collagen and Regenerate, with a compact mineral-like layer covering the lesion (Figure 6E). In the cross-sectional view, the minerals in the Regenerate and Sylc groups form a compact layer covering the outer edge of the treated lesion (Figure 7: D and E, respectively), masking the prisms and inter-prism structures. In contrast, in the collagen-treated group (Figure 7C), the surface appears smoother, and the prismatic structures are not easily recognized, thereby restoring the enamel prism defect after demineralization. The melting effect of CO_2_-treated surfaces can be observed from the surface view (Figure 6F), which appears irregular yet smoother than other treated surfaces. The outer edge of the treated surface appears smooth and homogenous in the cross-sectional view (Figure 7(F-1,F-2)), demonstrating complete coverage of the prismatic borders (Figure 7(F-3)).

## 4. Discussion

This study supported the effectiveness of various micro-invasive treatment approaches (bovine collagen supplement, Regenerate serum/toothpaste, Sylc air abrasion, and CO_2_ laser) in repairing enamel WSLs in controlled laboratory situations, as they increased the mineral content and tissue hardness of WSLs up to a depth of 150 µm. However, beyond this depth (200 µm), the values were comparable among all groups (*p* > 0.05); therefore, the stated hypothesis was partially rejected. The collagen supplement enhances the appearance and texture of treated lesions, showing areas of complete tissue repair within the lesion (Figure 5). The Regenerate and Sylc air abrasion techniques were reintegrated into the demineralized surfaces, forming a biomimetic layer that preserved the structure and morphology of WSLs, reducing their opacity and resulting in a glossier appearance. Exposure to the CO_2_ laser produced a profound enhancement in the appearance of WSLs, showing a glossy, smooth, and more polished surface, with a recognized reduction in their opacity. Raman spectroscopy is a precise technique that analyzes the molecular components in mineralized dental tissues, particularly the phosphate groups in hydroxyapatite, allowing for a quantitative assessment of tissue demineralization [27]. The Raman peaks associated with phosphate (P-O vibrations) in enamel are characterized by their wavenumber positions, intensities, and associated vibrational modes. The ν_1_ peak, which is the most intense, typically corresponds to the symmetric stretching vibrations of the phosphate groups and occurs at around 960–970 cm^−1^. The ν_2_ peak is linked to the bending vibrations of the phosphate groups at 430–440 cm^−1^, while the ν_4_ peak corresponds to the overtone or combination bands observed at 572 cm^−1^. These peaks signify the presence of phosphate-based crystalline minerals within enamel and are considered a quantitative measure for mineral loss or gain [28]. In the current study, all peaks are located in the same regions previously reported [6,29]. The dissolution of hydroxyapatite (HAp) crystals under acidic conditions produces a considerable reduction in the intensity of all phosphate peaks (ν_1_, ν_2_, and ν_4_) in the WSLs compared to all groups up to a depth of 150 µm (*p* < 0.000, Table 1). Similarly to the dynamic process of caries development, the H^+^ ions diffuse into enamel from the demineralizing solution, while calcium and phosphate ions are released, resulting in the dissolution of hydroxyapatite crystals in the intercrystalline and inter-prismatic spaces, which produces holes and cracks at the surface. This might explain the notable reduction in KHN values of WSLs up to a depth of 150 µm compared to all groups (*p* < 0.001, Figure 4). 

FE-SE micrographs (Figure 6 and Figure 7B) support this evidence, as the WSL reveals a disorganized, rough surface associated with higher prism exposure and porosity, along with a partial loss of the inter-prismatic substances. In contrast, the intact surface (baseline) is smooth and uniform (Figure 6A), exhibiting tightly fused enamel prisms and a less prominent prismatic structure, while the boundaries of the prisms and inter-prism gaps cannot be recognized in the cross-sectional views (Figure 7A). However, beyond 150 µm, the phosphate band intensities and KHN values were comparable across all surfaces (*p* > 0.05). This observation aligns with Al-Taee et al. (2022) [23], who reported that the depth of this lesion spans between 100 and 150 µm when measured by optical coherence tomography (OCT). Theoretically, the HAp crystals are tightly packed and well-organized in the superficial layer of enamel, contributing to higher tissue hardness, which gradually decreases towards the dentine–enamel junction (DEJ) [29]. However, in the current study, the phosphate content and KHN were found to be higher in the deeper layers. This may reflect enhanced mineral ion exchange and remodeling in the demineralized surfaces, which are porous and less mineralized superficially after exposure to acids. This allows for greater ion movement into the deeper layers, enhancing the redeposition of minerals back into the enamel throughout these layers, where the microstructure can become denser and more organized [30].

The structure and organization of collagen fibers vary among different tendon types due to differences in solubility and biochemical and biomechanical properties, which can be identified in FTIR spectral analysis. The collagen-containing structures exhibit the presence of the amide I band, with higher intensity indicating more densely packed collagen fibers comprising pyridinoline-type cross-linking, proline and/or hydroxyproline, and hydrogen bonding [26,31]. In the present study, the FTIR spectrum of bovine collagen supplement powder (Figure 3) showed the presence of ν (C=O) absorption at 1600–1700 cm^−1^, which correlates with the amide I, amide II, and amide III bands at 1540–1560 cm^−1^ and 1180–1300 cm^−1^, respectively, along with C-H band absorptions at 1350–1480 cm^−1^.Treating WSLs with a bovine collagen supplement resulted in a 50–130% enhancement in the phosphate peak intensities (ν_1_, ν_2_, and ν_4_) compared to untreated lesions (*p* > 0.001) at depths up to 150 µm. This enhancement was comparable to the baseline throughout the lesion depth and to the surfaces treated by the CO_2_ laser and BAG in the deeper layers (100–200 µm). These findings suggest that bovine collagen may have the ability to self-assemble on demineralized enamel surfaces, influenced by intermolecular hydrogen bonding and interactions from its side chains [32]. This can provide a scaffold that promotes the attachment of other proteins and minerals. This matrix provides binding sites with high affinity for Ca^2+^ ions, which act as a nucleator for the formation of de novo hydroxyapatite, ultimately leading to the remineralization of the lesion body [13]. In the presence of cations and a pH level below 7.4, it transforms into an elastomeric gel, resulting in the creation of a three-dimensional fibrillar network that promotes the biomineralization of the lesion [33]. However, the effectiveness of these proteins in promoting remineralization can vary depending on the quality of an individual’s saliva, particularly its pH, mineral content, and flow rate. Nevertheless, the results of the present study indicate the potential of the bovine collagen supplement to restore the mineral content in the demineralized surface, competing with the results of other methods that can form mineral complexes or change the crystalline structure of HAp to seal exposed lesions and improve surface integrity. This aligns with a previous study [34] that observed a notable increase in minerals in carious-like lesions after five days of treatment with a self-assembled peptide. The authors suggest that the peptide can create a framework to facilitate the deposition and growth of elongated enamel-like apatite, which has a higher packing density and is organized into interlocking enamel prisms when immersed in calcium and phosphate- containing solutions. Other studies [35,36] affirmed the remineralization ability of self-assembling peptides, which help regenerate hydroxyapatite crystals through the presence of saliva. Interestingly, the application of bovine collagen improved the hardness values by 60–110% throughout the entire depth of WSLs (50–200 μm), which was comparable to the baseline and other treatment approaches. This is in agreement with a previous study [35] that reported an enhancement in the mechanical properties of enamel when treated with self-assembled collagen to a depth of 125 μm, which is more effective than fluoride-based treatments. The exposed lesions appear well-covered when treated with collagen, as seen in the FESEM surface and cross-sectional views (Figure 6 and Figure 7C). In these images, the honeycomb-like structure and lacunae between enamel rods are not recognizable, which is associated with a reduction in the roughness and porosity of the treated lesion.

The highest PO_4_ (ν_1_, ν_2_, and ν_4_) intensity values among the groups were observed in the Regenerate-treated surfaces, which recorded a 70–160% increase in the phosphate content compared to untreated surfaces up to 150 µm (*p* < 0.001). This finding is consistent with previous studies [6,37] that supported the ability of calcium silicate-containing formulations to precipitate calcium phosphate crystalline phases on exposed enamel lesions. This is attributed to the release of Ca^2+^ ions, which exchange with hydrogen ions (H^+^) to form silanol compounds with silica (Si-OH). This silanol compounds act as a template, allowing salivary phosphate groups (PO_4_^3−^) to attract calcium ions and form a CaP-enriched layer that plays a vital role in remineralization. However, beyond a depth of 150 µm, the phosphate peaks intensities were comparable across all groups (*p* > 0.05). Furthermore, the availability of fluoride ions in these formulations may contribute to the re-hardening capacity of enamel and promote tissue remineralization. This could explain the increase in microhardness values (60–110%) in this group compared to WSLs up to a depth of 150 µm (*p* > 0.001). This aligns with another study [20] that noted improved hardness values in demineralized enamel surfaces following the application of MI Paste Plus, which contains calcium, phosphate, and fluoride ions that may permeate the porous surfaces to form mineral complexes, thereby facilitating tissue remineralization. The Regenerate-treated surface appears well-covered, with a notable reduction in the porosity of treated WSLs (Figure 6D). Moreover, the mineral deposits are distinctly visible when observed in cross-section (Figure 7(D-3)), where the prisms and inter-prism gaps are completely covered by mineral deposits along the prismatic borders.

The application of bioactive glass (BAG) via air abrasion produced a profound enhancement (33–148%) in the phosphate content (ν_1_, ν_2_, and ν_4_) and microhardness (60–110%) of treated WSLs throughout the lesions’ depth, comparable to other treatment groups in most layers (*p* > 0.05). The abrasion process selectively removes the superficial layers of WSLs, enabling the BAG particles to embed within the surface and providing a source of ions that promote tissue remineralization. 

Many in vitro studies [8,9,38,39] have reported enhanced surface properties of WSLs when treated with BAG as compared to untreated lesions. This is attributed to the ability of bioactive glass 45S5 (BAG) to form hydroxy-carbonate apatite (HCA) that can deposit within the exposed lesions, thus enhancing their mechanical properties. BAG consists of calcium, phosphate, and silicate ions that are released and exchanged with other ions in saliva. This interaction raises the interfacial pH, resulting in the formation of a silica-rich layer that acts as a matrix template for the precipitation of amorphous calcium phosphate, which subsequently crystallizes into HCA [38]. The BAG-treated surface is restored under FESEM, appearing smooth and homogenous, with minimal visibility of enamel prisms in the surface view (Figure 6E). Similarly in the cross-sectional view (Figure 7(E-3)), the prisms and inter-prism gaps are completely covered by a mineral deposit, restoring the surface integrity after demineralization.

The phosphate band intensity ν_1_ in the lased-treated surfaces was significantly higher than that of the non-treated WSLs and comparable to the baseline and all treated surfaces (*p* > 0.05). The mean intensity values of ν_2_ and ν_4_ were higher than those of the baseline and collagen-treated surfaces (*p* < 1.000), especially in the superficial layer (50 µm). This finding concurs with a previous study [6] that reported higher phosphate content in the CO_2_-irradiated surfaces compared to the baseline. This is attributed to the relatively low energy density (11.5 J/cm^2^) of the applied laser, which can be absorbed by minerals, inducing structural and chemical alterations in the HAp crystals. Following energy absorption, there is a rise in the temperature of the irradiated surfaces, which decomposes the organic matrix and reduces the carbonate content. This is followed by fusion and recrystallization into new crystalline phases, such as pyrophosphate, tri-calcium phosphate, or tetra-calcium phosphate, with a high calcium-to-phosphorus ratio [40]. These new crystals can seal the exposed WSLs and enhance the hardness of the irradiated surfaces up to a depth of 150 µm. This finding is consistent with Liu and Hsu, (2007) [40], Rodríguez-Vilchis et al., 2011 [41], and Garma and Jasim (2015) [42]. The melting and sealing effects of laser are observed in the FESEM (Figure 6F), where the surface appears textured but well-covered, with a significant reduction in roughness and porosity of treated lesions. In the cross-sectional view (Figure 7(F-1,F-2)), the lesion is restored, showing signs of mineral deposition at the surface layer. The intensity of the carbonate peak was measured by Raman spectroscopy at 1070 cm^−1^, which was the lowest in the CO_2_-irradiated surfaces, followed by the baseline and collagen-treated groups, up to a depth of 150 µm. This is due to the thermal effect of lasers, which decreases the water and carbonate levels in enamel, contributing to the reduction in solubility and thereby enhancing the enamel resistance to acids [6]. The preceding findings support the effectiveness of the bovine collagen supplement in treating early enamel lesions by restoring mineral content, tissue hardness, and surface morphology. These effects are similar to those observed with remineralization methods and the application of CO_2_ lasers when examined under controlled laboratory conditions. This treatment is particularly effective if the supplement is retained in the mouth for two minutes before swallowing or used as a mouthwash. The self-assembly mechanism of bovine collagen is still speculative; therefore, further investigations are needed, including molecular binding assays to determine the capability of bovine collagen to form a biomimetic scaffold, as well as immunohistochemistry tests and atomic force microscopy to confirm the structural integration of collagen with enamel. The artificial saliva is composed solely of inorganic ions, without consideration of the effect of salivary proteins, pellicles, and plaque on mineralization inhibition. Moreover, there is the possibility of experimental errors and dissimilarities in the micro-structure of enamel among specimens. The artificially created WSLs are typically induced under controlled laboratory settings within a specified timeframe. They produce lesions of homogeneous size and depth across samples, which differ from naturally occurring lesions caused by bacterial acids, exhibiting variations in depth, appearance, pore size, and degree of mineral loss. Although this model does not fully replicate the clinical scenario, it provides a foundational understanding of the potential mechanisms independent of the confounding variables present in the oral cavity. This approach is consistent with similar studies in the field that aim to establish proof of concept before progressing to more complex models. Although micro-invasive procedures using BAG air abrasion or a CO_2_ laser can offer benefits for managing enamel lesions, it is essential to consider their drawbacks and assess them in the context of each patient’s individual needs and circumstances. The air abrasion process can induce surface roughness, which may predispose to plaque accumulation and cause further decay if not appropriately managed. Additionally, the CO_2_ laser may create cracks and imperfections on the tooth surface that can potentially weaken the tooth structure over time. The success of these interventions is highly dependent on the operator’s experience. While the current study supports the effectiveness of these techniques for treating WSLs, uncertainty remains regarding their long-term outcomes when applied in clinical settings. Accordingly, patients may require additional treatment or follow-up care to achieve optimal results and prevent the recurrence of white spot lesions.

## 5. Conclusions

The current research suggests that the bovine collagen supplement can serve as a solution for managing early enamel lesions in vitro. This is achieved by the increased mineral content, tissue hardness, and enhanced structural integrity of the treated enamel lesions. This effect is similar to that achieved by the Regenerate system, Sylc air abrasion, and CO_2_ laser, all of which promote tissue remineralization and preserve the morphology and esthetics of enamel white spot lesions. 

## Figures and Tables

**Table 1:** Raman band intensities A.U. (Mean ± SD) of untreated baseline, WSLs, and the treated enamel surfaces (collagen, Regenerate, Sylc, and CO_2_ laser) at different depths 50–200 µm.

PO_4_ ν_1_	50 µm	100 µm	150 µm	200 µm	*p* Value
Baseline	1222.7 ± 77.6 ^a^	1322.6 ± 94.9 ^c^	1347.4 ± 89.0 ^d^	1352.7 ± 103.9 ^e^	0.051
WSLs	777.7 ± 73.4 *	880.8 ± 109.2 *	1014.6 ± 103.5 *	1290.5 ± 116.2 ^e^	0.000
Collagen	1226.5 ± 78.7 ^a^	1387.0 ± 94.3 ^c^	1360.2 ± 98.5 ^d^	1411.9 ± 98.0 ^e^	0.000
Regenerate	1340.1 ± 95.4 ^b^	1422.6 ± 92.6 ^c^	1372.4 ± 92.7 ^d^	1384.4 ± 78.0 ^e^	0.105
Sylc	1300.2 ± 86.4 ^ab^	1399.0 ± 97.7 ^c^	1350.0 ± 89.8 ^d^	1345.9 ± 80.4 ^e^	0.081
Laser	1268.5 ± 95.2 ^ab^	1341.3 ± 87.2 ^c^	1344.8 ± 93.6 ^d^	1359.3 ± 91.7 ^e^	0.053
**PO_4_ ν_2_**					
Baseline	195.7 ± 27.7 ^f^	243.6 ± 25.1	283.1 ± 22.0 ^i^	272.4 ± 28.1 ^j^	0.000
WSLs	132.8 ± 27.5 *	146.2 ± 23.0 *	154.5 ± 24.2 *	195.2 ± 24.8 *	0.000
Collagen	192.6 ± 35.1 ^f^	288.8 ± 22.4 ^h^	309.8 ± 24.9 ^i^	292.2 ± 28.8 ^j^	0.000
Regenerate	327.1 ± 37.7 ^g^	364.8 ± 24.0	402.0 ± 24.2	306.0 ± 25.8 ^j^	0.000
Sylc	313.6 ± 28.2 ^g^	327.7± 28.7 ^h^	305.5 ± 26.4 ^i^	284.4 ± 26.2 ^j^	0.051
Laser	302.0± 37.8 ^g^	308.8 ± 37.4 ^h^	308.2 ± 27.0 ^i^	290.1 ± 25.6 ^j^	0.490
**PO_4_ ν_4_**					
Baseline	164.3 ± 22.5 ^k^	168.1 ± 25.6 ^m^	230.5 ± 25.2 ^n^	260.5 ± 20.2 ^p^	0.000
WSLs	97.4 ± 20.9 *	100.2 ± 22.8 *	110.2 ± 22.8 *	187.7 ± 26.9 *	0.000
Collagen	161.4 ± 22.7 ^k^	182.8 ± 26.2 ^m^	252.6 ± 22.3 ^no^	286.1 ± 17.8 ^p^	0.000
Regenerate	266.8 ± 21.1	283.3 ± 28.3	310.9 ± 25.1	280.1 ± 18.9 ^p^	0.000
Sylc	217.4 ± 25.2 ^l^	249.3 ± 27.2	273.8 ± 23.9 ^o^	281.1 ± 30.0 ^p^	0.000
Laser	190.7 ± 22.9 ^l^	196.2 ± 22.5 ^m^	274.1 ± 18.6 ^o^	283.7 ± 27.5 ^p^	0.000
**Carbonate**					
Baseline	87.0 ± 20.1 ^r^	94.7 ± 25.2 ^tx^	96.2 ± 18.5 ^y^	99.8 ± 14.7 ^z^	0.552
WSLs	100.5 ± 23.8 ^r^	107.7 ± 18.1 ^t^	107.5 ± 21.1 ^y^	106.2 ± 23.0 ^z^	0.855
Collagen	88.6 ± 23.7 ^r^	86.8 ± 19.5 ^tx^	88.8 ± 20.6 ^y^	93.4 ± 20.1 ^z^	0.834
Regenerate	154.7 ± 18.3	166.7 ± 17.9	135.2 ± 22.4	104.5 ± 22.0 ^z^	0.000
Sylc	100.0 ± 24.2 ^r^	102.6 ± 18.4 ^t^	102.5 ± 16.7 ^y^	98.6 ± 19.2 ^z^	0.759
Laser	58.6 ± 19.9	73.3 ± 17.1 ^x^	84.7 ± 18.7 ^y^	99.3 ± 20.3 ^z^	0.005

(*) There are statistically significantly lower phosphate intensity values among groups. Similar letters indicate that there are no statistically significant differences between the groups at each depth (within each column). The *p* value refers to the presence of statistically significant differences in values between layers within each group, if the *p* value is less than 0.05. A multivariant ANOVA and Tukey’s post hoc tests were conducted at an alpha level of 0.05.

## Data Availability

The data that support the findings of the current study are available from the corresponding author upon reasonable request.
